# Cytotoxicity and Antibacterial Efficacy of AgCu and AgFe NanoAlloys: A Comparative Study

**DOI:** 10.3390/antibiotics11121737

**Published:** 2022-12-01

**Authors:** Fang Zhou, Elie Kostantin, De-Quan Yang, Edward Sacher

**Affiliations:** 1 Solmont Technology Wuxi Co., Ltd., Wuxi 214135, China; 2 CISSS de Laval, Hôpital Cité-de-la-Santé, 755 Bd René-Laennec, Laval, QC H7M 3L9, Canada; 3 Engineering School, Dali University, 2 Hongsheng Rd., Dali 671003, China; 4 Regroupement Québécois de Matériaux de Pointe, Département de Génie Physique, Polytechnique Montréal, Case Postale 6079, Succursale Centre-Ville, Montréal, QC H3C 3A7, Canada

**Keywords:** AgCu, AgFe, antibacterial efficacy, cytotoxicity, L929 cells, nanoalloy

## Abstract

Although Ag nanoparticles (NPs) have been widely applied in daily life and in biomedical and industrial fields, there is a demand for Ag-based bimetallic nanoalloys (NAs), such as AgCu and AgFe, due to their enhanced antibacterial efficacy and reduced Ag consumption. In this work, we present a comparison study on the antibacterial efficacy and cytotoxicity rates of Ag NPs and AgCu and AgFe NAs to L929 mouse fibroblast cells using the CCK-8 technique based on the relative cell viability. The concept of the minimum death concentration (MDC) is introduced to estimate the cytotoxicity to the cells. It is found that the minimum inhibitory concentrations (MICs) of the NPs against *E. coli* and *S. aureus* decrease with the addition of both Cu and Fe. There is a strong correlation between the MDC and MIC, implying that the mechanisms of both antibacterial efficacy and cytotoxicity are similar. The enhanced antibacterial efficacy to bacteria and cytotoxicity toward the cell are attributed to Ag^+^ release. The following order is found for both the MIC and MDC: AgFe < AgCu < Ag NPs. However, there is no cytotoxicity to the L929 cells for AgFe and AgCu NAs at their MIC Ag concentrations against *S. aureus*.

## 1. Introduction

The excessive use of antibacterial medications to counter even minor disorders has provoked evolutionary changes in the affected bacteria. Some of these changes have produced bacteria that are resistant to their standard medications, such as methicillin-resistant *Staphylococcus aureus* and vancomycin-resistant *Enterococcus*. Indeed, in a CDC (U.S. Department of Health and Human Services, Centers for Disease Control and Prevention) report [1], five bacteria and fungi are listed as urgent threats, eleven as serious threats, two as concerning threats and three as watch-listed. Undoubtedly, alternative therapies are needed, and metal-based nanoparticles (NPs) are potential candidates. For example, Ag NPs are some of the most vital and fascinating nanomaterials among the several metallic nanoparticles that are involved in the use of health care and biomedical devices to protect against viruses and bacteria [2,3]. However, their toxicity and antibacterial efficacy are still critical concerns that raise challenging issues for their application [2,4,5]. Bimetallic nanoalloys (NAs), e.g., AgCu and AgFe, are emerging as promising antibacterials, with their properties surpassing those of their monometallic counterparts. Two advantages can be presented for choosing these two NAs for practical applications—to reduce both the consumption of Ag and its attendant toxicity. Indeed, these NAs showed either enhanced antibacterial efficacy [6,7,8] or a reduced amount of Ag at their MIC concentrations. Although recent studies have emphasized the preparation of Ag NPs using biological (e.g., fungi) and plant (natural stabilizers and reductants) extracts in an effort to reduce toxicity [9,10], AgCu and AgFe NAs remain important candidates for reductions in toxicity to human cells and the enhancement of antibacterial efficacy.

However, there are some of exceptions. For example, Długosz et al. [11] reported that AgCu NAs prepared in a microwave reactor produced 27–97 nm Ag and Cu NPs and 32–184 nm AgCu NPs. There was no enhanced antibacterial effect compared with Ag or Cu NPs, although AgCu had no genotoxicity. For AgFe NAs, it was found that there was not only enhanced antibacterial efficacy, but also enhanced catalytic and antioxidant performance; in addition, reduced cytotoxicity was extensively reported. For example, Amendola et al. [12] found that polyethylene-glycol-coated AgFe NAs exhibited a high transverse reactivity (316 ± 13 mM^−1^s^−1^, >3 times that of the most common clinical benchmark based on iron oxide), excellent colloidal stability and biocompatibility in vivo; further, they were biodegradable within a few months. Mahmoudi et al. [13] reported that ultrathin (~1–2 nm) Ag ring-coated superparamagnetic iron oxide NPs (SPIONs) exhibited enhanced antimicrobial characteristics against bacteria while having no toxicity to human cells. Tao et al. [14] demonstrated that their AgFe graphene-based cellular monolithic (AgFe/GCM) materials possessed enhanced antibacterial activity and lower toxicity to human cells. Panchal et al. [15] prepared hierarchical AgFe coral-like NAs, which exhibited enhanced antibacterial efficacy against both Gram-positive and Gram-negative bacteria. Gonçalves et al. [16] reported that aminolevulinic-acid-coated AgFe NAs were effective against breast tumor cells (MCF-7 and LNCaP). Prucek et al. [17] showed that magnetic Ag-Fe_3_O_4_ and Ag-Fe_2_O_3_ NAs demonstrated enhanced antibacterial efficacy without showing toxicity to mice embryonal fibroblasts. Al-Asfar et al. [18] and Sandupatla et al. [19] demonstrated that AgFe NAs have excellent antibacterial, antiradical and antioxidant efficacy. However, some questions remain: (1) How does its antioxidant efficacy and catalytic performance affect its antibacterial efficacy and cytotoxicity? (2) How do the AgM (Cu or Fe) metals work together for enhanced antibacterial efficacy and reduced cytotoxicity?

In our recent review of the AgCu NP system [6], it became obvious that the efficacy rates were arranged in the following fashion: *Ag ~ Cu < (a mixture of Ag and Cu) < AgCu alloy*. Clearly, *Ag ~ Cu < (a mixture of Ag and Cu)* exhibits synergy, i.e., Ag and Cu operate through different additive mechanisms. However, why is it that *(a mixture of Ag and Cu) < AgCu alloy*? In this paper, the first of a series of articles on bimetallic NAs, we explore that question. AgFe has been specifically chosen for comparison with AgCu for several reasons:

- The atomic radii of Fe and Cu are similar;
- They are both first transition series elements;
- Both surfaces oxidize upon exposure to atmosphere [20,21];
- Both Cu and Fe are mutually immiscible with Ag;
- Cu has known antibacterial efficacy [6] while Fe has none [22].

It is through a comparison of the efficacy of these materials that we anticipate to obtain information on why *(a mixture of Ag and Cu or Fe) < AgCu or AgFe alloys*.

There appear to be three distinct methods with which the NA attacks the bacterium, although the exact mechanisms are in dispute:

- Contact killing, in which there is direct, ostensibly dry contact between the NP and bacterium. In fact, as recently noted [23], there is always a nanoscopic layer of water deposited on the NP surface from the ambient humidity. This serves to hydrolyze and ionize the NP surface oxide, which attacks the bacterium;
- Killing in liquids that support the ionization of the NP surface. This appears to be the case in many toxicological evaluations. The metal surface ionizes, leading to ionic diffusion. Ultimately, NPs from the metal are found in the outer membrane surfaces of the bacteria, as well as within the cytoplasm [6];
- Killing in liquids that do not support the ionization of the NP surface. This is the case for human body fluids, such as blood and lymph. They are highly saturated and also contain substances that can reduce metal ions to zero valency [24]. The attack of the bacterium occurs through the protein corona that forms about the NP [23].

The following question begs an answer: how representative are the cytotoxic values, determined in the second of these phases, of the antibacterial effects occurring in the first and third phases?

The cytotoxicity of metal-based NPs depends on their physicochemical properties [25,26], including the composition, size, shape, surface charge, solubility and aggregation of the NPs, as well as the coating materials and reactivity [27]. The coatings, such as stabilizers, are of special concern for toxicity evaluations. While it is commonly considered that biogenic capping agents offer the lowest toxicity for Ag NPs [28], their exact chemical structures have not been determined.

NP dispersions, made via metal salt reduction in aqueous solvents, are stabilized by the addition of polymeric substances (i.e., polyethylene glycol, PEG, polyvinyl pyrrolidone, PVP, polyvinyl alcohol, PVA, etc.) that surround the NP, preventing agglomeration and coalescence. Their adhesion to the NP surface is weak, and they can be removed by washing or by dilution, destabilizing the dispersion. We found that the use of a mixture of PVP and PVA stabilizes the dispersion to a greater degree [29], although the reason for the stabilization is not obvious. The coating not only prevents NP aggregation, but also affects the contact with the bacteria and cells, the Ag^+^ release, the new NP formation and the chemical reduction. More detailed information on this topic can be found in a recent review [6].

PVP has been shown to interact with metal atoms on the NP surface through the formation of a coordinate covalent bond using the lone pair on the carbonyl O [30]. Concentrated aqueous mixtures of PVP and PVA, when subjected to microwaves, crosslink covalently, ultimately forming a hydrogel [31]. However, dilute mixtures, such as used here, are known to interact through hydrogen bonding [32].

In the present work, we compare the antibacterial efficacy and cytotoxicity of AgCu and AgFe NAs, based on our previous papers [33,34,35], prepared in the same manner and using the same stabilizers, so as to eliminate the effect of the changing physicochemical properties on the cytotoxicity and antibacterial efficacy [25,26]. Therefore, their antibacterial efficacy and cytotoxicity are comparable, showing similar trends, whereby both MIC and MDC (minimum death concentration) follow the order of *Ag NPs > AgCu NAs > AgFe NAs*. We offer explanations as to why this is so. Our intention in this work is to focus on the correlation between the antibacterial efficacy and cytotoxicity, rather than on their preparation and physicochemistry, which were addressed in our previous publications [33,34,35].

## 2. Results

**Figure 1:**
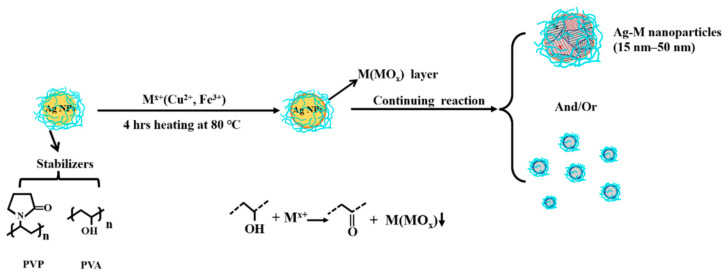
Schematic of the silver-based bimetal NA preparation using Ag NP catalysis.

AgM NAs were obtained via the Ag NP catalysis of M ions at 80 °C for 4 h, as illustrated in Figure 1. Briefly, the AgM NAs are formed through the following processes. (1) The initial PVP-PVA-stabilized AgNPs catalyze the M ions and reduce them on the Ag NP surface at elevated temperatures [33] and then deposit M on the Ag NP surface to form metal layers, (2) causing the formation of AgM NAs; then, (3) these AgM NAs coalesce and reorganize into larger AgM NAs. In addition, (4) excess metal ions are reduced directly to M or MO_x_ NPs.

**Figure 2:**
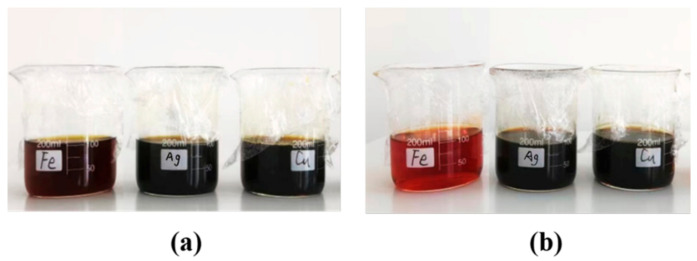
Photographs of Ag, AgCu and AgFe NAs before (**a**) and after (**b**) heating at 80 °C for 4 h. The Ag concentration was 500 ppm for all the samples. The labels Fe, Ag and Cu represent AgFe, Ag and AgCu, respectively.

**Figure 3:**
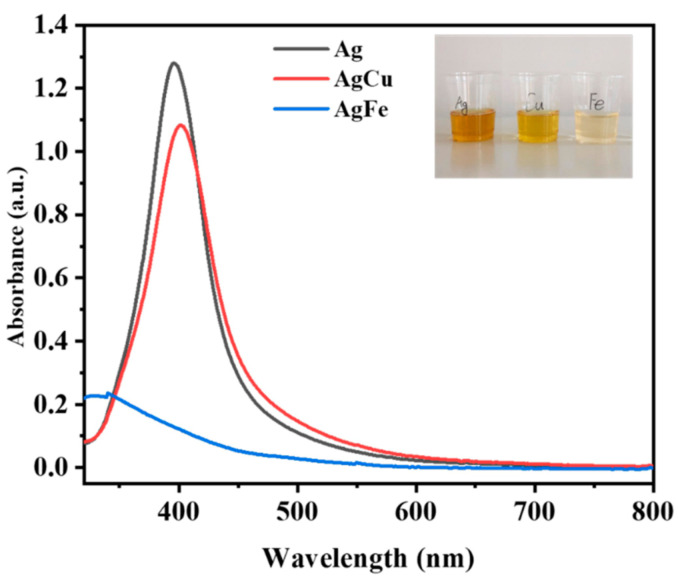
A comparison of the UV–visible spectra of Ag, AgCu and AgFe. The Ag concentration was 10 ppm for all samples and the Ag/M ratio was 2:1. The inset shows a color comparison of the AgNPs, AgCu NAs and AgFe NAs at the 10 ppm Ag concentration.

The appearances of all samples containing 500 ppm of Ag can be found in Figure 2. All appeared to be dark yellow, both before and after heating, except for the AgFe samples, which changed to orange/reddish after heating. We confirmed that all samples were alloyed using techniques from our previously study [33]. Their UV–visible spectra are displayed in Figure 3, containing 10 ppm of Ag. The figure insert shows the color comparison of Ag and AgM at 10 ppm. It reveals that the yellow color gradually lessened from Ag to AgCu to AgFe. At the same time, the Ag SPR peak disappeared for AgFe, while it only decreased for AgCu, and its peak position shifted from 396 nm to 405 nm. The UV-Vis comparison is only shown for an Ag/M ratio of 2:1. The variations in SPR values based on the Ag/M ratio can be found in our related papers [33,36].

**Figure 4:**
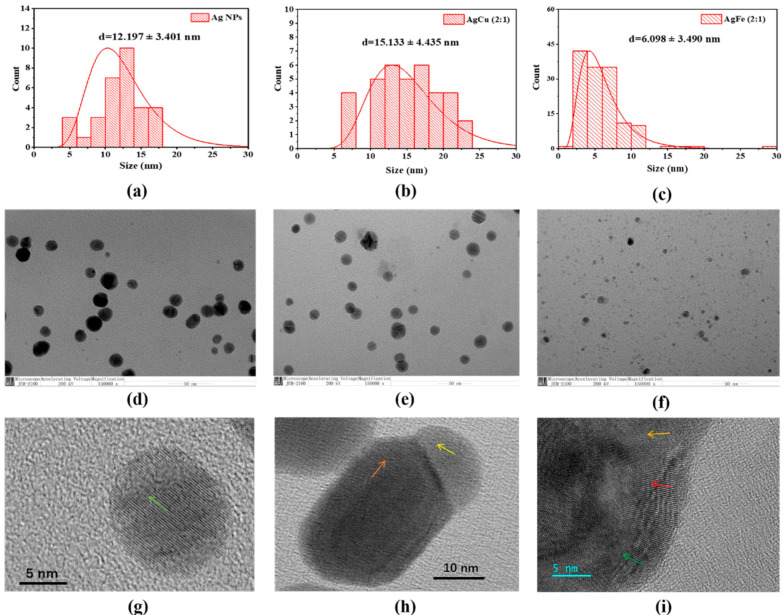
Size distributions and TEM photomicrographs of (**a**,**d**,**g**) Ag, (**b**,**e**,**h**) AgCu and (**c**,**f**,**i**) AgFe NAs. The arrows indicate local atomic lattice orientations.

Figure 4 contains the typical morphologies of the AgM NAs (Figure 4d–f) and their HR-TEM photomicrographs (Figure 4g–i); these indicate that the Ag NPs are single crystals (Figure 4g) and AgM are alloyed (Figure 4h,i). The average sizes of the NAs, based on the size distribution in Figure 4a–c, increased compared with the Ag NPs. The AgFe NAs show a log-normal distribution. Figure 4f shows many small Fe-contained NPs, which XRD and XPS analyses have shown to be γ-Fe_2_O_3_ [36].

**Figure 5:**
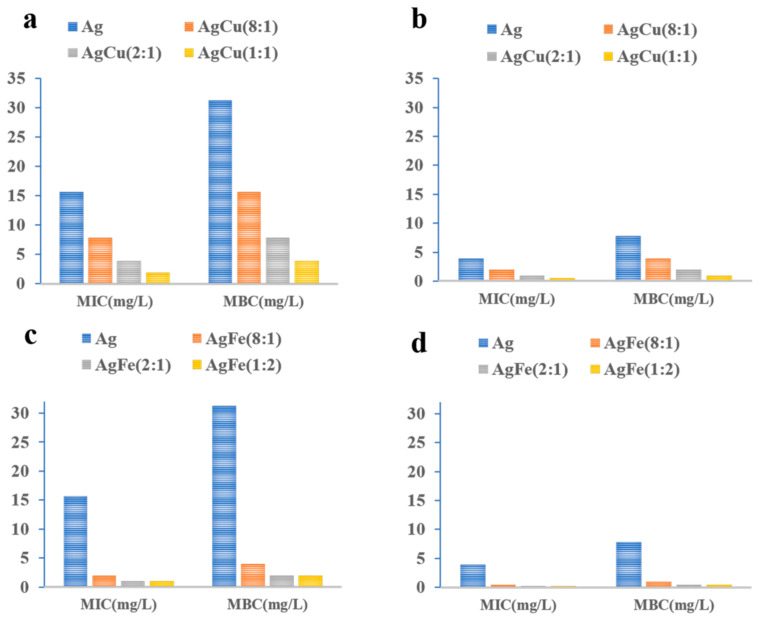
MIC/MBC comparison of various Ag/M ratios against (**a**,**c**) *S. aureus* (ATCC 6538) and (**b**,**d**) *E. coli* (ATCC 8099).

Figure 5 shows the MIC/MBC comparison of the Ag, AgCu and AgFe with different Ag/M ratios against *S. aureus* and *E. coli*. The standard deviations are absent in Figure 5 because of the experimental method used for the MIC values in Section 4.4. Both the MIC and MBC values become smaller with increasing Cu and Fe concentrations for the two strains. For a given Ag concentration, the order of efficacy is *Ag NPs < AgCu NAs < AgFe NAs*. In addition, we explored the antibacterial effects of two commercial antibiotics, as shown in Table S1 in the Supplementary Materials, to compare them with Ag-based nanomaterials. The order of the efficacy is *Ag NPs < AgCu NAs < AgFe NAs* ≈ ampicillin < ciprofloxacin for *S. aureus*; and *Ag NPs < AgCu NA*s ≈ ampicillin *< AgFe NA*s ≈ ciprofloxacin for *E. coli.*

**Figure 6:**
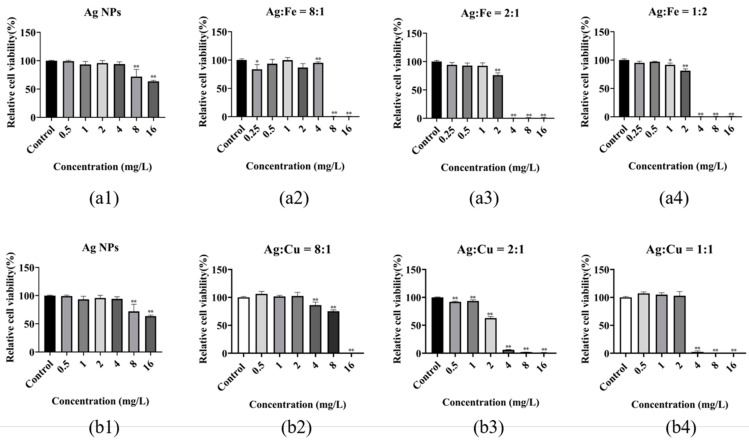
Concentration-dependent cytotoxicity of various Ag/M ratios. (**a1**,**b1**) for Ag NPs, (**a2**–**a4**) for AgFe NAs and (**b2**–**b4**) for AgCu NAs. Note: Compared to the control, * *p* < 0.05; ** *p* < 0.01.

Figure 6 shows the Ag-concentration-dependent cytotoxicity of AgM NAs as a function of the Ag/M ratio. The toxicity can be seen to be Ag-concentration-dependent, with the relative cell viability rates slowly decreasing with the increasing Ag concentration, before abruptly decreasing at ~60%. It seems from Figure 6 that the greater the M (Cu or Fe) fraction, the lesser the Ag concentration at the abrupt drop point. At a constant Ag concentration, increasing the M ratio leads to an increase in toxicity to the cell. The order of cytotoxicity is *Ag NPs < AgCu NAs < AgFe NAs* for a given Ag concentration, which is similar to their antibacterial activities.

## 3. Discussion

The color changes seen in Figure 2 (at a higher Ag concentration of 500 ppm) and in the insert in Figure 3 (at a lower Ag concentration of 10 ppm), and confirmed by the changes in SPR peak intensities [33] (Figure 3) and the TEM data in Figure 4, indicate the formation of AgM NAs. As shown previously, Ag NPs measuring 12.2 ± 3.4 nm in average size, based on the TEM observations, change to 15.1 ± 4.4 nm and 6.1 ± 3.5 nm for AgCu and AgFe, respectively, confirming the schematic depicted in Figure 4. The log-normal distribution of the AgFe NAs in Figure 4c can be caused by the formation of small Fe_2_O_3_ NPs. The antibacterial efficacy rates of AgCu and AgFe NAs for both *E. coli* and *S. aureus* (Figure 5) are greatly enhanced over those of Ag NPs, with both the MIC and MBC values of AgFe being superior to those of AgCu. None of this can be explained by the Ag/M synergy.

**Figure 7:**
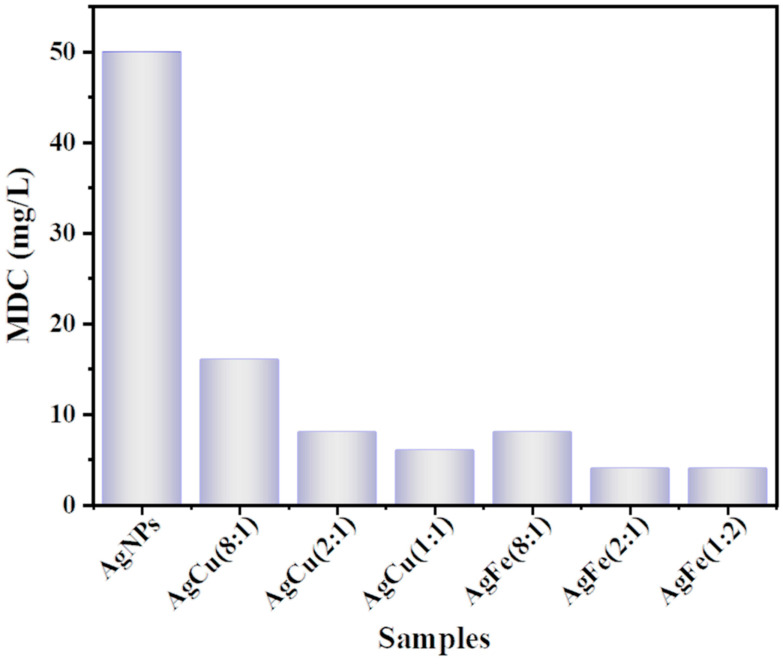
MDC values for various Ag/M ratios.

**Figure 8:**
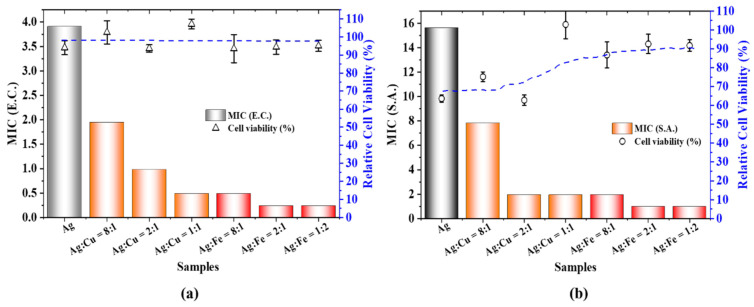
A comparison of the MICs and relative cell viability rates of Ag, AgCu and AgFe NAs for (**a**) *E. coli* and (**b**) *S. aureus.*

Although AgFe and AgCu NAs are more cytotoxic than Ag NPs at the same Ag concentration (Figure 6), the abrupt drops in the relative viability rates indicated that the Ag concentration is critical. In order to better understand the cytotoxicity, we define the minimum Ag concentration at complete death as the minimum death concentration (MDC), which is the lowest concentration of the agent that reduces the viability of the initial cell inoculum by a pre-determined value, such as ≥99.0%. This gives us the MDC variations for the samples, as shown in Figure 7, which are similar to the MIC variations in Figure 8. The relative cell viability changes of the samples are also shown in Figure 8, indicating the absence of toxicity at the MIC for *E. coli* (Figure 8a), the absence of toxicity for all Ag/Fe ratios and with the exception of Ag/Cu = 1 or less for *S. aureus* at the MIC (Figure 8b), based on ISO standard 10993, where a reduction in cell viability by more than 30% is considered a cytotoxic effect.

**Figure 9:**
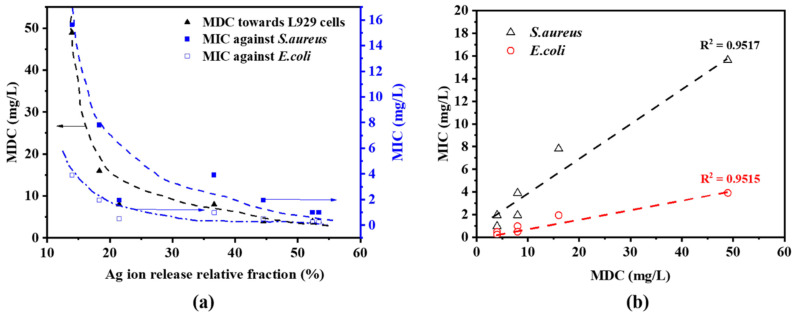
(**a**) MIC and MDC variations with released Ag^+^ fractions for Ag, AgCu and AgFe. (**b**) MIC values as a function of the MDC values for Ag, AgCu and AgFe.

The Ag^+^ release from AgM NAs is a major reason [37,38,39,40] for the antibacterial mechanism. We assessed the Ag^+^ concentrations in Ag, AgFe and AgCu. The dependences of the MIC and MDC on the Ag^+^ concentration are plotted in Figure 9a. Clearly, the greater the Ag^+^ concentration, the greater the antibacterial efficacy (lower MIC), implying that Ag^+^ plays a key role. The plots of both the MIC and MDC values follow only the Ag ion concentration in Figure 9a for both *E. coli* and *S. aureus*. The enhancement of the antibacterial efficacy appears greater for *S. aureus* than for *E. coli* (Figure 5 and Figure 9a), indicating that the AgM NAs are more effective for Gram-positive bacteria. This is caused by the fact that Gram-negative bacteria are surrounded by a lipid bilayer, whose outer leaf contains lipopolysaccharides; these include polysaccharide chains extending outward from the surface of the bilayer [41]. It is generally accepted that their presence blocks the approach of the drugs to the membrane and is the reason why Gram-negative illnesses are more difficult to treat.

The antibacterial comparison between the Ag/AgM NPs and commercial antibiotics in Figure 5 and Table S1 inspired us to explore more efficient nano-antibiotics as substitutes for the treatment of infections, and AgFe NAs perform well in this case.

The MDC dependences in Figure 9a is plotted against the MIC dependences in Figure 9b. They are linearly correlated with the MDCs for the two strains and the L549 cells with a R^2^ value of ~0.95, suggesting that the toxicity mechanisms are similar. In addition, Figure 9b suggests that the correlation between MIC and MDC values is independent of the sample, whether for AgFe or AgCu.

**Figure 10:**
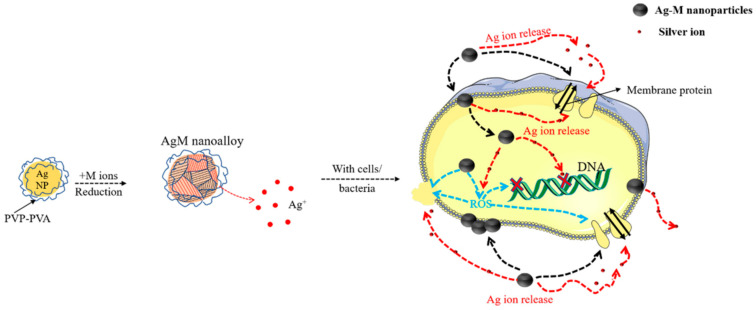
The proposed toxicity mechanism of AgM NAs.

It is well known that the antibacterial efficacy and cytotoxicity mechanisms for the Ag NPs and AgM NAs are associated with interactions of the NP surface with the cell or bacterium. These include the following interactions: (1) Ag^+^ release in the media, as Ag^+^ can directly interact with the cell, generating reactive oxygen species (ROS) and inducing oxidative stress and inflammation, which consequently damage the cell membrane, proteins and DNA [42,43,44]. Oxidative stress is considered to be the probable mechanism of Ag NP-induced toxicity, involving superoxide (O_2_^−^) and H_2_O_2_. (2) NP–cell interactions and cellular uptake, whereby the NPs pass through the cell wall, penetrating into the cell [45,46,47], causing cell wall damage and leakage of cytoplasm. Based on this, we propose the mechanism in Figure 10. The main process is the release of Ag^+^, which can penetrate into the bacterial cell and react with proteins or produce excess ROS; all of these interactions can induce cell death. Both AgCu and AgFe are able to release more Ag^+^, which enhances the antibacterial efficacy compared with Ag NPs. Although both the antibacterial activity and cytotoxicity are affected by size [25,48,49,50], this should be a mild effect, as in our case the NAs sizes are similar (except for γ-Fe_2_O_3_, which is not toxic) and the size in this range is not sensitive to cell toxicity [48,49,50].

An additional factor may be at play here, namely that antioxidants are able to reduce their cytotoxicity [51,52,53]. Although these studies showed that the introduction of additional antioxidants, such as ascorbic acid (vitamin C) and quercetin, can reduce the toxicity of Ag NPs, there is no report on the relationship between the NP cytotoxicity and antioxidant performance. As Figure S1 in the Supplementary Materials section indicates, we found that there were different antioxidant activities among the NPs in the order of AgFe > AgCu > Ag for a given Ag concentration. This will offset the NP toxicity to the cell, and may be the reason for the cytotoxicity order of AgFe < AgCu < Ag at their MIC Ag concentrations. While there is much evidence to support the occurrence ROS-related toxicity, Monprasi et al. [54] recently showed that a significant increase in intracellular ROS, induced by AgCu NAs, did not cause THP-1 cell death, consistent with the previous research results [55,56]; that is, factors besides ROS may also affect the cytotoxicity. For example, Ag ion release from AgM NAs can cause oxidative stress, resulting in increased toxicity to the cell, while antioxidants of the AgM NAs will decrease the production of ROS through scavenging free radicals [57], as with the introduction of ascorbic acid. This means that the antioxidant action of the AgM NAs is one of reasons for reducing the cytotoxicity, which is in need of a systematical study in the future.

## 4. Materials and Methods

### 4.1. Materials

A commercially available aqueous dispersion of 1000 ppm (*w*/*v*) Ag NPs, stabilized with a mixture of PVP and PVA, was provided by Solmont Technology Wuxi Co., Ltd. (Wuxi, China). The copper nitrate (Aladdin, Shanghai, China, AR grade, CAS No 10031-43-3) and ferric nitrate nonahydrate (InnoChem, Beijing, China, CAS No 7782-61-8) were used as received. Milli Q water was used to dilute the suspensions to the desired concentration. The Costar 96-Well Clear Flat Bottom plates were obtained from Corning, Inc. Ciprofloxacin (Aladdin, ≥ 98%, CAS No 85721-33-1) and ampicillin trihydrate (Aladdin, 96%, CAS No 7177-48-2) are two broad-spectrum antibiotics used for antibacterial testing.

*Staphylococcus aureus* (ATCC 6538) and *Escherichia coli* (ATCC 8099), obtained from the China Center of Industrial Culture Collection, were utilized to evaluate the antibacterial effects of the NPs. The L929 mouse fibroblast cell line was obtained from Shanghai iCell Bioscience Inc., (Shanghai, China).

### 4.2. Synthesis of AgM NAs

The Ag NP dispersion was diluted to 500 ppm with Milli Q water, and several concentrations of aqueous cupric or ferric solution were added to obtain AgCu and AgFe NAs with ratios such as 8:1 and 2:1. The Cu/Fe ions were adsorbed onto the Ag NP surface upon heating the dispersion in an oven at 80 °C for 4 h. The specific parameters of the samples with different precursor ratios can be found in Table 1. The concentrations indicated in the article are those used for Ag.

### 4.3. Characterizations

The UV–visible absorption spectra were obtained on a Yoke 723N (Yoke Instruments Co., Ltd., Shanghai, China) UV–visible absorption spectrophotometer. The sizes, shapes and crystallinities of the NAs were determined on a TEM system (JEM 2100, JEOL, Tokyo, Japan), and an HR-TEM system (JEM-F200, JEOL, Tokyo, Japan).

### 4.4. Antibacterial Efficacy (Minimum Inhibitory Concentration Tests)

The antibacterial efficacy rates of the NPs were evaluated against Gram-negative *Escherichia coli* and Gram-positive *Staphylococcus aureus*. The sub-culture of the bacterial colony was made directly from the primary culture. The bacteria were grown overnight on a nutrient agar media plate. The inoculums of 0.5 McFarland standards (1.5 × 10^8^ CFU/mL) were maintained in nutrient broth by picking up a single colony from the sub-culture plate [7].

The MIC (minimum inhibitory concentration) tests were carried out using the broth dilution method [58]. Several NA dilutions were made in sterile distilled water, and 100 μL of each dilution was applied to 96-well plates containing 100 μL of bacteria at 10^6^ CFU/mL, which were then cultured at 37 °C overnight. Before and after incubation, the MIC was determined via visual observations and validated via turbidity measurements [59]. Each assay was repeated at least 3 times. Finally, the MIC value was determined via the situation under which the minimal concentration of the antibacterial agent inhibited the growth of the bacteria three times or more, which meant that MIC results for a sample were exactly the same and the standard deviation was zero.

### 4.5. Cytotoxicity

The cytotoxic efficacy toward L929 mouse fibroblast cells, by means of the Cell-Counting-Kit-8 (CCK-8) method, were conducted as follows. First, 6 × 10^3^ cells per well were placed into 96-well plates and incubated for 24 h at 37 °C under 5% carbon dioxide. The cells were then treated with different nanomaterial concentrations for 24 h. The culture medium containing 10% CCK-8 was added into each well and cultured at 37 °C under 5% carbon dioxide for 2 h. The viability was detected at 450 nm using the ELISA spectrophotometer [60]. The results for all iterative tests are provided as the means ± SD, with *p* < 0.05 denoting a significant difference.

### 4.6. ICP-MS Analysis

In order to study the release abilities of Ag^+^ in the NAs, the samples were centrifuged at 12,000 rpm for 30 min. For each 1 mL of supernatant obtained, 4 mL of 10% HNO_3_ was added to acidify the solution for analysis [61,62]. The concentrations of Ag^+^ in the solution were measured via inductively coupled plasma mass spectrometry (ICP-MS, Agilent 8800, Santa Clara, CA, USA). Metal ions were detected three times for each sample and the results for all iterative tests are provided as the means ± SD.

### 4.7. Antioxidation Activities

The antiradical activities were determined using the nitrogen-centered stable radical 2,2-diphenyl-1-picrylhydrazyl (DPPH) scavenging method [63,64]. The decrease in absorbance of the reaction mixture (DPPH solution + AgM NAs) was measured at 517 nm via UV–visible spectrophotometry. In a typical experiment, 6 mg of DPPH was added to anhydrous ethanol to a volume of 200 mL. A DPPH solution of the same concentration, in the absence of AgM NAs, was used as the control and the AgM NAs in anhydrous ethanol solution at the same concentration were used as a blank. The reaction mixture was incubated in the dark at room temperature for 30 min. Each sample and the control were analyzed at least three times. The DPPH inhibition rate was calculated using Equation (1):

(1) $$\mathrm{DPPH}\;\mathrm{scavenging}\;\mathrm{effect}\;(\%)=\frac{A_{3}-\left(A_{1}-A_{2}\right)}{A_{3}}\times 100\%$$

where *A*_1_ = the absorbance of the sample (absorbance of DPPH solution + samples), *A*_2_ = the absorbance of the blank (absorbance of samples + deionized water) and *A*_3_ = the absorbance of the control (absorbance of DPPH solution + deionized water).

## 5. Conclusions

We studied both the antibacterial activity and cytotoxicity rate for an Ag/AgCu/AgFe system with the same stabilizers and preparation process, using *S*. *aureus*, *E. coli* and mouse fibroblast L929 cells. Our study reveals that the antibacterial efficacy of AgCu and AgFe are greatly enhanced over that of Ag, with the order for both the antibacterial efficacy and cytotoxicity being *AgFe > AgCu > Ag* for a given Ag concentration. More importantly, these AgM NAs may be possible antibiotic substitutes. Although there was a substantial enhancement of the antibacterial efficacy of the AgM NAs compared to Ag NPs, there was no measurable cytotoxicity for AgM NAs at their MIC Ag concentration. The main mechanism of both the antibacterial activity and cytotoxicity appears to be Ag^+^ release, although antioxidant activity may also play a part.

## Figures and Tables

**Table 1:** Parameters of the NA preparations.

Samples	Ag NPs	M Ions	Ratio of Ag/M Ions	Heating (80 °C)	Heating Time
Ag	500 ppm	0	-	No	-
AgCu_1_	500 ppm	62.5 ppm	8:1	Yes	4 h
AgCu_2_	500 ppm	250 ppm	2:1	Yes	4 h
AgCu_3_	500 ppm	500 ppm	1:1	Yes	4 h
AgFe_1_	500 ppm	62.5 ppm	8:1	Yes	4 h
AgFe_2_	500 ppm	250 ppm	2:1	Yes	4 h
AgFe_3_	500 ppm	1000 ppm	1:2	Yes	4 h

## Data Availability

Not applicable.

## Supplementary Materials

The following supporting information can be downloaded at: https://www.mdpi.com/article/10.3390/antibiotics11121737/s1. Table S1. MIC/MBC rates of two commercial antibiotics against two strains. Figure S1: Ag ratio to metal (M=Cu (a) and Fe (b)) concentration based on the antioxidant properties of AgM NPs.
