# Supplementary material for: Cytotoxicity and Antibacterial Efficacy of AgCu and AgFe NanoAlloys: A Comparative Study
Source: Antibiotics (Basel). 2022 Dec 1;11(12):1737. doi: 10.3390/antibiotics11121737 (PMC9774506; doi:10.3390/antibiotics11121737)
Supplement: Supplementary file 1 [file antibiotics-11-01737-s001.zip › antibiotics-2017545-supplementary.pdf]

Supplementary Materials

Table S1. MIC/MBC of two commercial antibiotics against two strains.

Antibiotics	*S. aureus* (ATCC 6538)		*E. coli* (ATCC 8099)	
	MIC (mg/L)	MBC (mg/L)	MIC (mg/L)	MBC (mg/L)
Ampicillin	0.7813	1.5625	1.5625	3.125
Ciprofloxacin	0.1953	0.3906	0.3125	0.625

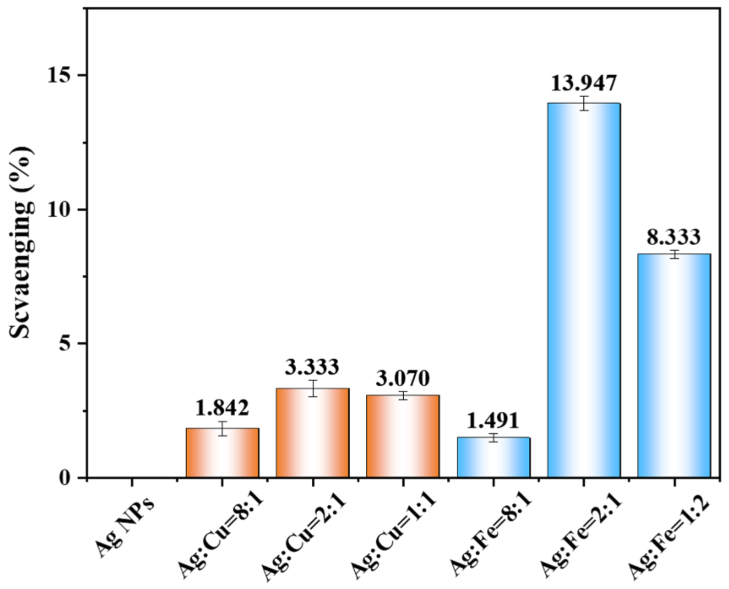

Figure S1. Ag ratio to metal (M=Cu(a) and Fe(b)) concentration on antioxidant properties of AgM NPs.
